# Metastatic organotropism: a brief overview

**DOI:** 10.3389/fonc.2024.1358786

**Published:** 2024-04-25

**Authors:** Margarida Carrolo, João A. I. Miranda, Guilherme Vilhais, António Quintela, Mário Fontes e Sousa, Diogo Alpuim Costa, Francisco R. Pinto

**Affiliations:** ^1^Hematology and Oncology Department, CUF Oncologia, Lisbon, Portugal; ^2^BioISI – Institute for Biosystems and Integrative Sciences, Faculty of Sciences, University of Lisbon, Lisbon, Portugal; ^3^Medical Oncology Department, Hospital de Cascais, Cascais, Portugal; ^4^NOVA Medical School, Faculdade de Ciências Médicas, Lisbon, Portugal

**Keywords:** metastasis, organotropism, pre-metastatic niche, immune response to metastasis, brain metastasis

## Abstract

Organotropism has been known since 1889, yet this vital component of metastasis has predominantly stayed elusive. This mini-review gives an overview of the current understanding of the underlying mechanisms of organotropism and metastases development by focusing on the formation of the pre-metastatic niche, immune defenses against metastases, and genomic alterations associated with organotropism. The particular case of brain metastases is also addressed, as well as the impact of organotropism in cancer therapy. The limited comprehension of the factors behind organotropism underscores the necessity for efficient strategies and treatments to manage metastases.

## Introduction

Metastasis is the process by which cancer cells leave their primary site of tumorigenesis and colonize distant sites. Unlike primary tumors, which can be potentially treated with local therapies, metastasis is a systemic disease and remains the leading cause of morbidity and mortality among cancer patients (1). Yet, the process of metastasis is highly inefficient. Tumors can release millions of cells daily, and certain tumors initiate the dissemination of cells at an early stage in their development (2). Establishing colonization post-extravasation constitutes the major bottleneck in the metastatic progression, with fewer than 0.02% of cells succeeding in generating macroscopic metastases (2, 3).

Different cancer types exhibit organ-specific patterns of metastasis: gastric, gallbladder, pancreatic, and colorectal cancers frequently form established liver metastases before spreading to secondary sites like the lungs. Breast cancers commonly metastasize to the lung, bone, brain, and liver, while prostate cancer primarily metastasizes to bone (4, 5). Blood flow patterns influence the dispersion of circulating tumor cells (CTCs) in the body. The liver and lungs are often the first organs CTCs encounter, justifying why these organs are common locations for metastasis in various types of cancer. Liver and bone marrow sinusoids gaps promote the extravasation of CTCs and play a role in the increased prevalence of liver and bone metastases (2). Nevertheless, anatomical characteristics can only provide a partial explanation for organotropism. So, how do we explain the nonrandom pattern of cancer metastasis? Research to solve this topic has been divided into two main areas: molecular and cellular factors that influence organotropism (how it happens), and genetic differences in cancer cells that determine organotropism patterns (why it happens). There is however a gap in the understanding of the connection between the two areas. We hope that by providing a summary of both perspectives, this mini-review will motivate efforts to close this gap.

## Surviving bloodstream migration and conditioning the pre-metastatic niche

Before reaching a metastatic niche, CTCs need to survive during migration through the bloodstream. Tumor cells can circulate in the bloodstream either through individual cell dissemination or, more favorably, through collective migration. When tumor cells spread in clusters, distinct variations in function, morphology, and gene expression emerge among the cells. Outer cells, involved in interactions with the surrounding environment, display a notable degree of plasticity and mesenchymal characteristics, while inner cells maintain epithelial traits (6). Tumor cell clusters associate with platelets through selectins to evade harm caused by exposure to hemodynamic shear forces (7, 8). Regardless of their eventual metastatic destination, all CTCs must endure migration through the bloodstream. The characteristics necessary to survive bloodstream migration are probably unrelated to organotropic metastatic patterns. Yet, the molecular signals emanating from CTCs and the primary tumor, such as tumor derived factors and tumor derived exosomes, can influence distinct organs and tissues in different ways, resulting in fluctuations in the success rate of metastasis across various locations.

The pre-metastatic niche (PMN), a concept devised by Kaplan and colleagues in 2005, corresponds to a tissue microenvironment that provides support and the necessary conditions for the survival and proliferation of tumor cells (9). In contrast to the previous idea that distant organs were passively receptive to metastatic cells, numerous studies have suggested the existence of a tumor secretome, which produces factors, including hormones, chemokines, growth factors, and extracellular vesicles (EVs) that are secreted into the circulation, leading to the preconditioning of the future metastatic site (10–12). The PMN displays organ specificity but holds shared features within metastatic niches. PMNs are characterized by the induction of immunosuppression to avoid detecting metastasizing cells by tissue-resident T cells and natural killer (NK) cells and the promotion of inflammation with cytokines that regulate tumor growth. Other common features are the stimulation of angiogenesis and vascular permeability to enhance the infiltration of the tissue stroma by CTCs, the recruiting of bone marrow-derived cells (BMDC) and other non-resident cells that release factors that attract CTCs, and the remodeling of the extracellular matrix (ECM) to facilitate the adhesion of CTCs and BMDCs (9, 13).

These PMN pro-metastatic alterations are carried out by local stromal cells activated by tumor-derived signals, such as cancer-associated fibroblasts (CAFs). These can be found both within the primary tumor microenvironment and the PMN, known for providing mechanical support for tumor cells and playing crucial roles throughout the metastatic cascade (14). CAFs and their EVs also promote the formation of the PMN, through the secretion of chemokines, cytokines and ECM components, metabolic remodeling, immunosuppression and angiogenesis, thereby creating a favorable microenvironment at the future metastatic site (15). These effects appear to be organ specific, as show in a murine model of salivary adenoid cystic carcinoma, where primary tumor CAF EVs were specifically uptaken by lung fibroblasts, inducing the formation of a PMN and promoting the formation of lung metastases (16).

Conditions that allow the survival of a metastasizing cell when arriving at a particular PMN might not be ideal to promote proliferation. So, they might enter a phase of proliferative quiescence known as protective dormancy. Tumor cells can remain as dormant single cells or in micrometastatic clusters, in some cases for up to decades (17). Induction of dormancy is encouraged by growth-inhibitory signals present in the PMN, such as transforming growth factor-beta (18). Crucial intercellular interactions contribute significantly to the promotion of emergence from dormancy. Interactions that could potentially trigger emergence from dormancy include the activation of Wnt and Notch signaling, integrin-mediated cell signaling, and cell-adhesion interactions through the L1 cell adhesion molecule (19, 20).

## Immune defenses against metastases

The idea of an intricate interaction between tumors and immune cells traces back to the initial observation of Rudolf Virchow that leukocytes could infiltrate tumors (21). William Coley, noticing that a patient with an inoperable sarcoma was cured after an infection, proposed that the immune system had an anti-tumor role (22). Each organ owns a range of mechanisms for immune surveillance that constitute a primary defense against tumor cells. These mechanisms are organ-specific and influence the rate of success of metastatic cells (23).

Tumor cells produce antigens, known as tumor-associated antigens, which ideally should be recognized by the immune system. Like all other nucleated cells, tumor cells, present their antigens to Cluster of Differentiation 8 positive (CD8^+^) T lymphocytes via Major Histocompatibility Complex class I (MHC-I) molecules. Professional antigen presenting cells (APCs), like dendritic cells (DCs), capture tumor-associated antigens. DCs become activated, migrate to secondary lymphoid organs and present tumor-associated antigens on MHC-I and MHC-II molecules to CD8^+^ and CD4^+^ T cells, respectively. If T cells are sufficiently activated by DCs, they migrate to the tumor tissue and initiate cancer cell death upon recognizing their target antigen (22).

Various preclinical and clinical findings substantiate the theory that the immune system regulates certain tumors. For instance, mice deficient in B and T cells exhibit a higher occurrence and accelerated growth of induced tumors (24, 25). Correlational studies have demonstrated a link between T cell tumor infiltration and patient survival across various cancer types, such as breast, ovarian, and colorectal cancers (26–28). Clinical and experimental observations suggest that the adaptive immune system inhibits metastasis formation. For example, the depletion of CD8^+^ T cells resulted in increased lung and reproductive tract metastases in a melanoma mouse model (29). Effective immune surveillance has the potential to eliminate tumor cells. However, immune responses against cancer are often compromised in individuals with cancer. Commonly, tumor cells downregulate MHC-I molecules to evade antigen presentation. Also, tumor cells upregulate the expression of immune checkpoints such as programmed death-ligand 1 (PD-L1), that usually block immune responses against self-antigens. Furthermore, tumor-derived factors are known to interfere with the maturation of DCs, inducing immature phenotypes that are less prone to activate T cell anti-tumor responses (22, 30).

Besides DCs, other innate immunity cells can influence metastatic functions. Lack of NK cells increases the risk of metastases in mice (31). Furthermore, the presence of metastasis was negatively correlated with the number of circulating or tumor-infiltrating NK cells in several solid tumors, including colorectal and gastric cancer (32, 33). Neutrophils and macrophages can promote cancer cell death through processes such as the release of cytokines, the generation of reactive oxygen species, and phagocytosis. These immune cells can also contribute indirectly by facilitating the recruitment of T cells into the tumor by producing chemokines (34). But these cells can also be regulated by tumor-derived signals and contribute to tumor and metastasis growth by promoting inflammation, ECM remodeling, angiogenesis and immunosuppression (35, 36). These tumor-regulated cells can mediate organotropism patterns. In breast cancer, G-CSF expanded neutrophils facilitated lung metastasis by suppressing NK cell anti-tumor activity (37). Patients with early stage breast cancer that had serum neutrophil extracellular traps (NETs) were more likely to develop liver metastasis (38). In pancreatic cancer, liver resident macrophages were shown to uptake tumor derived exosomes. This led them to secrete transforming growth factor beta (TGF-β) which upregulated fibronectin production by hepatic stellate cells, promoting liver metastasis formation (39).

External factors that influence immune cell populations at a given tissue can also affect metastasis formation at that location. An *in vivo* murine model study recently showed that an anaerobic bacteria, *Fusobacterium nucleatum*, can promote liver metastasis in colorectal cancer by reshaping the hepatic metastatic immune microenvironment. *F. nucleatum*-treated mice exhibited elevated peripheral and hepatic myeloid-derived suppressor cell infiltration but decreased NK cells, as well as CD3^+^, CD4^+^, and CD8^+^ T cells in the liver as compared to control mice (40). Accordingly, another study has also demonstrated that this microorganism can be detected in liver metastases and that *F. nucleatum* positive colorectal liver metastases are associated with reduced T-cell density (41).

## The particular case of brain metastases

The occurrence of organotropism in the brain is a complex phenomenon governed by interactions between metastatic cancer cells and the microenvironment of brain tumors. The prognosis is generally unfavorable for brain metastases, regardless of their primary tumor origin. Melanoma, breast, and lung cancers exhibit higher rates of brain metastases when compared to other cancer types (42). Nevertheless, the reasons why certain cancers metastasize to the brain more frequently than others remain somewhat unclear. Cancer cells face significant challenges in infiltrating the brain due to the formidable blood-brain barrier characterized by tightly interconnected endothelial cells (42). Nevertheless, many patients present with metastases in the brain but not the liver (5). Specific tumor cells possess a distinct array of mechanisms enabling them to breach the blood-brain barrier, selectively establishing themselves in the brain.

Several studies indicate that the brain-specific affinity exhibited by particular cancer types is determined by a synergistic blend of capabilities within specific genetic subtypes of primary tumors or their subclones. These capabilities include detachment, dissemination, and the ability to penetrate the blood-brain barrier (42). Moreover, brain metastasis tumor cells showed low levels of oxidative stress related genes in their transcriptome, suggesting an adaptation to the brain metabolic environment (43). Additionally, brain metastases can present mutations that are not found in the primary tumor, lymph nodes, or even other metastases of the same patient (44). Notably, numerous mutations of this nature represent clinically actionable targets such as Human Epidermal growth factor Receptor 2 (ERBB2), proto-oncogene B-Raf (BRAF), Master Regulator of Cell Cycle Entry and Proliferative Metabolism (MYC), and Breast Cancer gene 2 (BRCA2), potentially paving the way for targeted therapeutic approaches.

## Genomic alterations associated with metastasis organotropism

Genomic alterations that may influence organotropic behaviors of metastatic cancer cells can be acquired either during primary tumor development or during metastasis cell spread and colonization of distant organs (45). Large-scale sequencing studies have tried to identify genomic alterations associated with metastases by comparing the genomic sequences of cohorts of metastases versus cohorts of primary tumors, including multiple cancer types (46–48) or cancer type-specific studies (49, 50). A paired comparison of metastases genomes with their matching primary tumor genomes has shown that most cancer-driver gene mutations have already occurred in the primary tumor (51–53). Genomic alterations associated with metastasis are hard to detect, probably because they may differ according to the cancer type and specific metastasis location (45). Besides the previously referred brain metastases-specific mutations (44), analyses of metastatic breast cancer samples according to metastasis site have identified significant associations of some mutated driver genes with specific metastasis locations (54, 55). Recently, a study comparatively analyzed the genomic sequences of both primary tumor and metastases samples of a large pan-cancer cohort of more than 25,000 patients, most of whom have metastatic disease (56). The large sample size allowed the identification of more than 50 genomic alterations associated with metastasis to specific target organs within sub-cohorts of individual cancer types.

Besides genomic alterations, gene expression signatures associated with specific metastases target organs have been identified (57–59). Some of these signatures influence the intercellular communication between tumor cells and stromal/immune cells in the target organ. In contrast, others allow the metabolic adaptation of metastatic cells to the target organ. It remains unclear how the organotropic patterns associated with genomic alterations are related to gene expression changes that facilitate metastases at specific locations. It may also be true that some of these gene expression signatures are characteristic of the original primary tumor cell types, even before the alterations induced by tumorigenesis.

## Organotropism and therapeutics for metastatic cancers

With the current therapeutic options, advanced metastatic disease is, with few exceptions, incurable (22). Understanding the mechanisms that contribute to metastasis organotropism patterns may suggest new treatment strategies to specifically block metastasis formation. Edelfosine, an ether lipid with anti-tumor activity, was shown to inhibit lung and brain metastasis in mice experiments. This lipid reorganizes membrane lipid rafts inhibiting adhesion to type I collagen and laminin 1 substrates (60). The adaptation of tumor cells to a new environmental niche includes metabolic reprogramming. By doing so, tumor cells will gain access to the available metabolites favoring its survival. As a result, directing therapeutic efforts towards the tissue specific metabolism of metastases could prove to be an effective approach for treating metastatic cancer (61, 62). Cancer cells with phosphatidylinositol-5-phosphate 4-kinase type 2 gamma (Pip4k2c) loss increased liver metastasis potential because they became hypersensitized to insulin, an abundant stimulus in this organ (63). Therefore, pharmacologically blocking insulin signaling can reset the advantage of these cells in liver colonization.

Immunotherapy is a treatment option for some metastatic tumors, improving patients prognosis (64). Immune checkpoint inhibitors (ICIs), such as anti-CTLA-4, anti-PD-L1 and anti-PD-1 are among the most successful immunotherapies. ICIs work as negative regulators of T cells, playing a crucial role in preventing tumor cells suppression of T cell activation (65). Effective immunotherapies for metastases must consider factors such as the location and type of tumor since these influence local immune cell populations. For instance, emerging data from anti-PD-1 clinical trials suggest that immune checkpoint inhibition offers greater benefits for patients with lung metastases compared to those with liver metastases (66). Within patients with liver metastasis receiving ICIs, prognosis varies according to the origin of the primary tumor. Urinary system tumors had the worst prognosis, while the efficacy of ICIs was less affected in digestive system tumors with liver metastasis (67). Within the immunotherapy arsenal, chimeric antigen receptor (CAR) T cell therapy has already proven to successfully treat hematopoietic malignancies. Nevertheless, this success has proven challenging to replicate in solid tumors, mainly due to the lack of tumor-associated antigen targets and an immunosuppressive tumor microenvironment (68). Metastases may exhibit differences from their primary tumors in terms of mutational and immune profiles. Also, the immune landscape across metastases originating from the same primary tumor may not be uniform within the same patient leading to potential paradoxical responses to therapy (69). Success of all immunotherapies depends on the cooperative activity of resident immune cells at the metastatic site. As previously discussed, cancer cells recruit and reprogram several innate immunity cell types to hamper proper anti-metastatic immune responses. To counteract this, inducing trained immunity (TI) has been proposed as a valid coadjuvant therapeutic approach. TI refers to an enhanced functional state of innate immunity cells resulting from exposure to microbial stimuli recognized by pattern recognition receptors such as dectin-1 or NOD2. TI is reached through long-lasting epigenetic reprogramming at the level of histone methylation and acetylation. The TI enhanced state favors anti-tumor immune responses and appears to be more insensitive to tumor-derived signals (70).

## Conclusion

**Figure 1:**
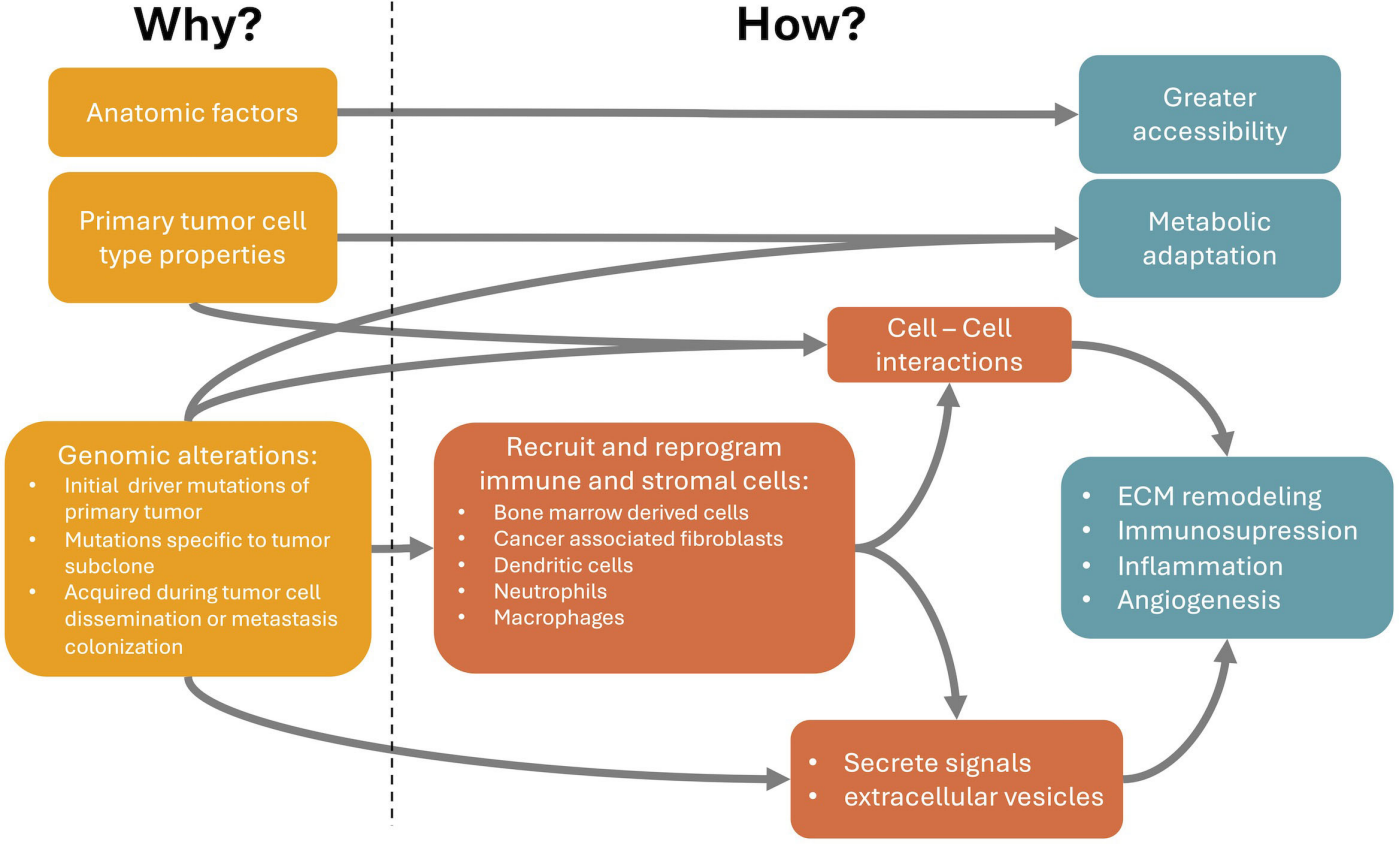
Relationship between the causes (Why it happens) and the cellular and molecular mechanisms of metastasis organotropism (How it happens). Anatomic relations between primary tumor and metastasis locations partially explain organotropism patterns through a facilitated accessibility of circulating tumor cells (CTCs) to particular metastatic locations. Properties of the primary tumor cell type, even before changes induced by tumorigenesis, can already explain a better metabolic adaptation or intercellular communication at the preferred metastatic niches. Finally, genomic alterations, such as mutations and copy number variations, can occur in the initial primary tumor development, subsequently in specific subclones of the primary tumor or are only acquired during tumor cell dissemination or metastasis colonization. These genomic alterations can modify cancer cell properties and favor specific metastatic locations through the secretion of tumor-derived signals or extracellular vesicles that can recruit and reprogram immune and stromal cells at the metastatic site, even before the arrival of CTCs. These reprogrammed cells can be, for example, bone marrow derived cells, cancer associated fibroblasts, dendritic cells, neutrophils or macrophages. They become pro-metastatic and induce processes like extracellular matrix (ECM) remodeling, immunosuppression, inflammation and angiogenesis. These processes facilitate the arrival and survival of CTCs at the metastatic niche.

The nonrandom metastasis patterns result from a cross-talk between cancer cells and distant organs. The primary tumor selectively and actively modifies organs of future metastasis before metastatic spread, potentially allowing the opportunity to halt the process. Understanding organotropic metastasis is a path for the development of novel treatment strategies. For that we need to close the gap between the causes of metastasis organotropism and the cellular and molecular mechanisms underlying it (**Figure 1**). In this review we present a brief summary of the mechanisms that explain how organotropism patterns arise and of genomic alterations associated with these patterns. The latter may be the ultimate cause of organotropic patterns. However, the genomic alterations with known associations with organotropism patterns are relatively scarce. This can be due to technical difficulties in demonstrating these associations. Alternatively, other causes of organotropism, such as anatomical factors and other properties of primary tumor cell types (**Figure 1**), may have a higher influence in the generation of organotropism patterns. On the other hand, there is a broader understanding of cellular and molecular mechanisms involved in metastasis organotropism. This knowledge can support the development of new therapies against metastatic disease.

## Author contributions

MC: Conceptualization, Investigation, Methodology, Validation, Visualization, Writing – original draft, Writing – review & editing. JM: Conceptualization, Investigation, Methodology, Validation, Visualization, Writing – original draft, Writing – review & editing. GV: Writing – original draft, Writing – review & editing. AQ: Supervision, Writing – original draft, Writing – review & editing. MS: Supervision, Writing – original draft, Writing – review & editing. DC: Supervision, Writing – original draft, Writing – review & editing. FP: Conceptualization, Supervision, Writing – original draft, Writing – review & editing.
